# Structured request form in musculoskeletal radiology examinations (CONCERTO): results of an expert Delphi consensus—structured radiology request form for correct classification of patients to undergo radiological examinations of the Italian Society of Medical and Interventional Radiology (SIRM), the Italian Society of Rheumatology (SIR) and the Italian Society of Orthopedics and Traumatology (SIOT)

**DOI:** 10.1007/s11547-024-01762-6

**Published:** 2024-02-05

**Authors:** Fausto Salaffi, Maria Antonietta Mazzei, Alberto Aliprandi, Fabio Martino, Biagio Moretti, Enzo Silvestri, Nunzia Di Meglio, Giulio Bagnacci, Marco Di Carlo, Luigi Sinigaglia, Roberto Gerli, Paolo Tranquilli Leali, Carlo Faletti, Andrea Giovagnoni

**Affiliations:** 1 Rheumatology Clinic, Università Politecnica delle Marche, “Carlo Urbani” Hospital, Via Aldo Moro, 25, 60035 Jesi, Ancona, Italy; 2 Unit of Diagnostic Imaging, Department of Medical, Surgical and Neurosciences, Department of Radiological Sciences, University of Siena, Azienda Ospedaliera Universitaria Senese, Siena, Italy; 3 Unit of Radiology, Clinical Institutes Zucchi, Via Bartolomeo Zucchi, 24, 20052 Monza, Italy; 4 Sant’Agata Radiology Center, Bari, Italy; 5 Department of Translational Biomedicine and Neuroscience (“DibraiN”)-Operative unit of Orthopaedics and Traumatology, University General Hospital, Bari, Italy; 6 Diagnostica per Immagini, Istituto Salus-Alliance Medical, Genoa, Italy; 7 Specialista in Reumatologia e Medicina Interna, Casa di Cura “La Madonnina”, Milan, Italy; 8 Department of Medicine, Rheumatology Unit, University of Perugia, Perugia, Italy; 9 Orthopaedic Department, University of Sassari, Sassari, Italy; 10 Dipartimento per Immagini dell’A.O. C.T.O-C.R.F.- M, Turin, Italy; 11 Department of Radiology, University Hospital “Azienda Ospedaliera Universitaria delle Marche”, Ancona, Italy

**Keywords:** Delphi method, Structured radiology request form, Consensus, Musculoskeletal diseases

## Abstract

**Purpose:**

To describe a Delphi consensus for the realization of a structured radiology request form for patients undergoing musculoskeletal imaging.

**Methods:**

A steering committee (four radiologists, a rheumatologist and an orthopedic surgeon) proposed a form to an expert panel (30 members, ten radiologists, ten rheumatologists and ten orthopedic surgeons). Through an online survey, the panelists voted on their level of agreement with the statements of the form using a 10-point Likert scale (1: no agreement; 10: total agreement) in a three-round process. A combination of two distinct criteria, a mean agreement level ≥ 8 and a percentage of at least 75% of responses with a value ≥ 8, was deemed as acceptable.

**Results:**

The form achieved high median ratings in all the assessed key features. During the first round, all items met the threshold to be advanced as unmodified in the next round. Additional proposed items were considered and introduced in the next round (six items in Section 1, five items in Section 2, ten items in Section 3, 11 items in Section 4, six items in Section 5, eight items in Section 6, ten items in Section 7 and eight items in Section 8). Of these items, in round 3, only six reached the threshold to be integrated into the final form.

**Conclusions:**

Implementation of a structured radiology request form can improve appropriateness and collaboration between clinicians and radiologists in musculoskeletal imaging.

## Introduction

Radiology request forms serve as essential communication tools utilized by healthcare institutions and medical practitioners to facilitate the referral of patients for radiological examinations. However, their significance often goes unrecognized. Inadequate clinical information or unrealistic assumptions about the capabilities of radiological methods can lead to unclear or ineffective communication between referring physicians and radiologists [1, 2]. In modern medical practice, there is an increasing reliance on dependable clinical laboratory and radiological services [3]. Diagnostic errors resulting from suboptimal request forms can lead to increased costs and unnecessary fatalities [4].

Radiologists have identified several shortcomings in these forms, including inaccurate selection of imaging procedures and therapies, insufficient patient history or details, vague clinical inquiries, a lack of standardized terminology and unclear acronyms. Additionally, challenges such as difficulties in contacting referring providers by phone and patient prioritization issues have been reported. Radiologists have suggested improving interactions with physicians and implementing training programs as potential solutions to these challenges [5].

Radiology request forms are considered both clinical and legal documents, typically completed by a referring physician or an authorized representative. These forms play a pivotal role in conveying the specific procedure needed and the underlying justifications for it [6]. It is advisable to complete this request form thoroughly and legibly in accordance with the guidelines published by the Royal College of Radiologists to reduce the risk of misinterpretation [7, 8]. According to relevant articles in the Radiation Protection Regulations of European Union Nations [9, 10], the referring physician is responsible for gathering all diagnostic information justifying the requested radiological examinations and documenting any previous exposures.

A comparison between the American College of Radiology and the Royal College of Radiologists reveals that a radiology request form should include the following information: clinical context, the question to be addressed, the patient’s personal details (name, age, address, and telephone number), the specific ward where the patient is located, the name and signature of the requesting physician, the identity of the consultant overseeing the patient’s care, and the date of the document. Despite this, radiologists receive minimal formal instruction regarding the interpretation of radiological requests and their significance as legally binding medical documents [8, 11].

This article presents the findings of a study that employed the expert Delphi Consensus methodology to develop a structured radiology request form. The study involved Italian radiologists, rheumatologists, and orthopedic surgeons, with the aim of establishing a standardized approach for accurately categorizing patients requiring radiographic investigations for musculoskeletal conditions.

## Methods

### Writing committee, panel composition and general structure of the request form

Initially, a six-member writing committee composed of four radiologists (M.A.M., C.F., F.M. and E.S.), a rheumatologist (F.S.), and an orthopedic surgeon (B.M.), all with decades of experience in the diagnosis of musculoskeletal diseases, proposed a form to be filled in for the correct classification of patients to undergo radiological examinations. This first form was organized into eight different sections according to the clinical and diagnostic phase of an appropriate assessment for musculoskeletal diseases: (1) patient personal information, four items; (2) pathologic and pharmacologic history, two items; (3) anatomical region to be explored, one item; (4) clinical features (fever, pain), four items; (5) trauma history, two items; (6) joint effusion information (swelling, synovial fluid analysis), three items; (7) previous local treatments (joint infiltrations, surgery), two items; and (8) clinical question and type of radiological examinations, two items. The 20 points were discussed by the writing committee both via email and in face-to-face meetings until a consensus agreement was reached.

### Questionnaires and Delphi process

A team of coordinators, consisting of three radiologists with experience in consensus development processes [11] (M.A.M., G.B., and N.D.M.), conducted the Delphi method following the current guidelines [12, 13].

The steering committee then invited a taskforce of 30 participants, equally distributed between ten radiologists, ten rheumatologists and ten orthopedic surgeons, selected from the most experienced members on this topic in the Italian Society of Medical and Interventional Radiology (SIRM), the Italian Society of Rheumatology (SIR) and the Italian Society of Orthopedics and Traumatology (SIOT), respectively. Invitations were individually emailed to selected participants, and anonymity was maintained throughout the Delphi process.

According to the literature, conducting a Delphi process requires a minimum of 12 experts. The decision to set a minimum of ten experts per group was made with the feasibility of recruiting genuine experts and ensuring group balance in mind. Including inexperienced participants and creating unbalanced groups might have introduced bias into the results. All the radiologists involved have extensive experience in diagnosing musculoskeletal diseases and were selected through a rigorous process within the SIRM. Furthermore, all the radiologists either currently work in, or have previously worked in, specialized hospitals focused on musculoskeletal disorders.

The positive response rate to adhesions was 100% (30 out of 30 experts). The Delphi process is a group facilitation technique that involves a multi-step iteration with the goal of transforming individuals’ opinions into group consensus [11–15]. Members of the writing committee did not attend the Delphi process.

The Delphi process was conducted in three different rounds. Questionnaires were sent out, and the experts’ responses were evaluated and shared anonymously with the writing committee and coordinators after each round. The coordinators also set thresholds to achieve adequate consensus for proposed items and to include additional suggestions in a statement in the next round (Table 1). Items that did not reach adequate consensus during a round were revised based on the free comments or additional suggestions proposed in the same round by the panel and then re-proposed for voting in the next round.

**Table 1:** Threshold established to evaluate the items during round iteration

Fixed threshold	Round 1	Round 2	Round 3	Action
Item	Median ≥ 8 IQR ≤ 2	Median ≥ 8 IQR ≤ 2	Median ≥ 8 IQR ≤ 2	Preserve the item
	Median < 8 IQR > 2	Median < 8 IQR > 2	Median < 8 IQR > 2	Remove the item
Additional suggestion	≥ 16/30 votes	≥ 16/30 votes	≥ 16/30 votes	Include into the item
	≤ 15/30 votes	≤ 15/30 votes	≤ 15/30 votes	Remove

*IQR* interquartile range

**Fig. 1:**
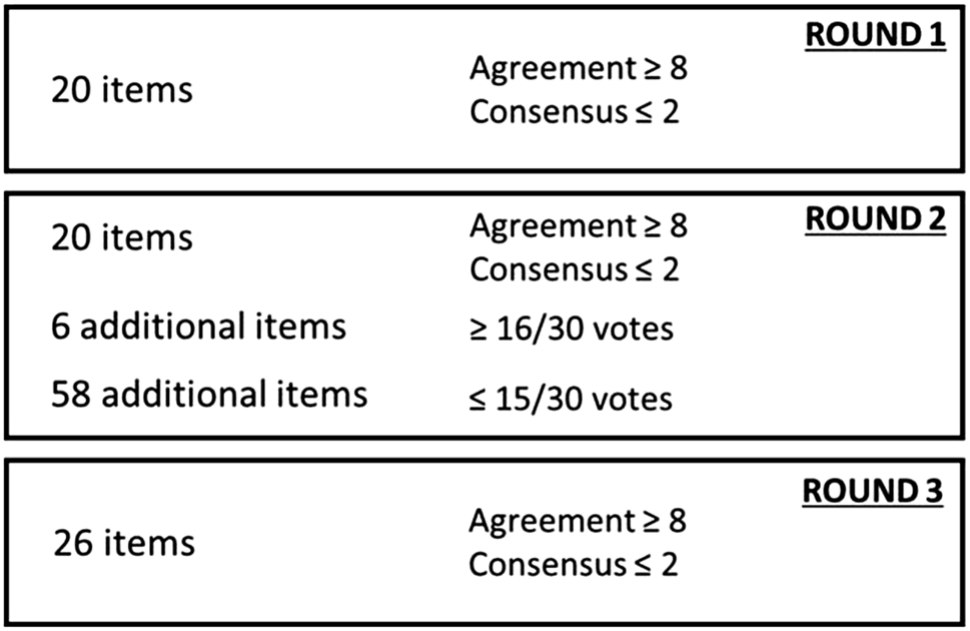
Delphi iterations

Each round was administered through the Google Form survey platform. Questionnaires were submitted with a maximum response period of 30 days and a 30-day interval between response collection and the next round. Figure 1 shows a detailed overview of how the Delphi rounds, and iterations were organized and managed.

In round 1, the expert panel evaluated the original statements formulated by the writing committee using a Likert scale ranging from 1 to 10. In this round, the possibility to propose additional suggestions and add free comments were also given to the members of the expert panel.

The additional suggestions proposed by the expert panel in round 1 were taken into account by the writing committee when creating the items for round 2. Items that achieved adequate consensus during round 2 (≥ 16 votes) were introduced in the forms; conversely, additional suggestions that achieved a low consensus were removed. In rounds 2 and 3, an agreement scale ranging from 1 to 10 was adopted.

### Statistical analysis

The Delphi rounds were conducted using a 10-point Likert scale. Data were analyzed regarding consensus, agreement, and stability in all rounds. Consensus is defined as a degree of inter-expert agreement, and it is expressed as the interquartile range (IQR). In contrast, agreement, expressed as a median, is intended as the degree of agreement with statements. Lastly, stability, defined as the consistency of subjects’ responses in successive rounds, was assessed by Wilcoxon matched-pairs signed-rank test (with *p* values < 0.05 indicative of no stability).

## Results

The structured request form obtained elevated median ratings in all key characteristics assessed. The response rate of the group of experts in the first and second rounds was 100%. In round 3, the response rate reduced to 96.7% (29/30), with nine out of ten rheumatologists responding.

During round 1, all items reached the threshold to be advanced as unmodified in the next round with a high agreement (item medians range 8.5–10) and a sufficient consensus (IQR range 1–2). Some additional proposed items from the expert panel were considered and introduced in the next round (six items in Section 1, five items in Section 2, ten items in Section 3, 11 items in Section 4, six items in Section 5, eight items in Section 6, ten items in Section 7 and eight items in Section 8).

**Fig. 2:**
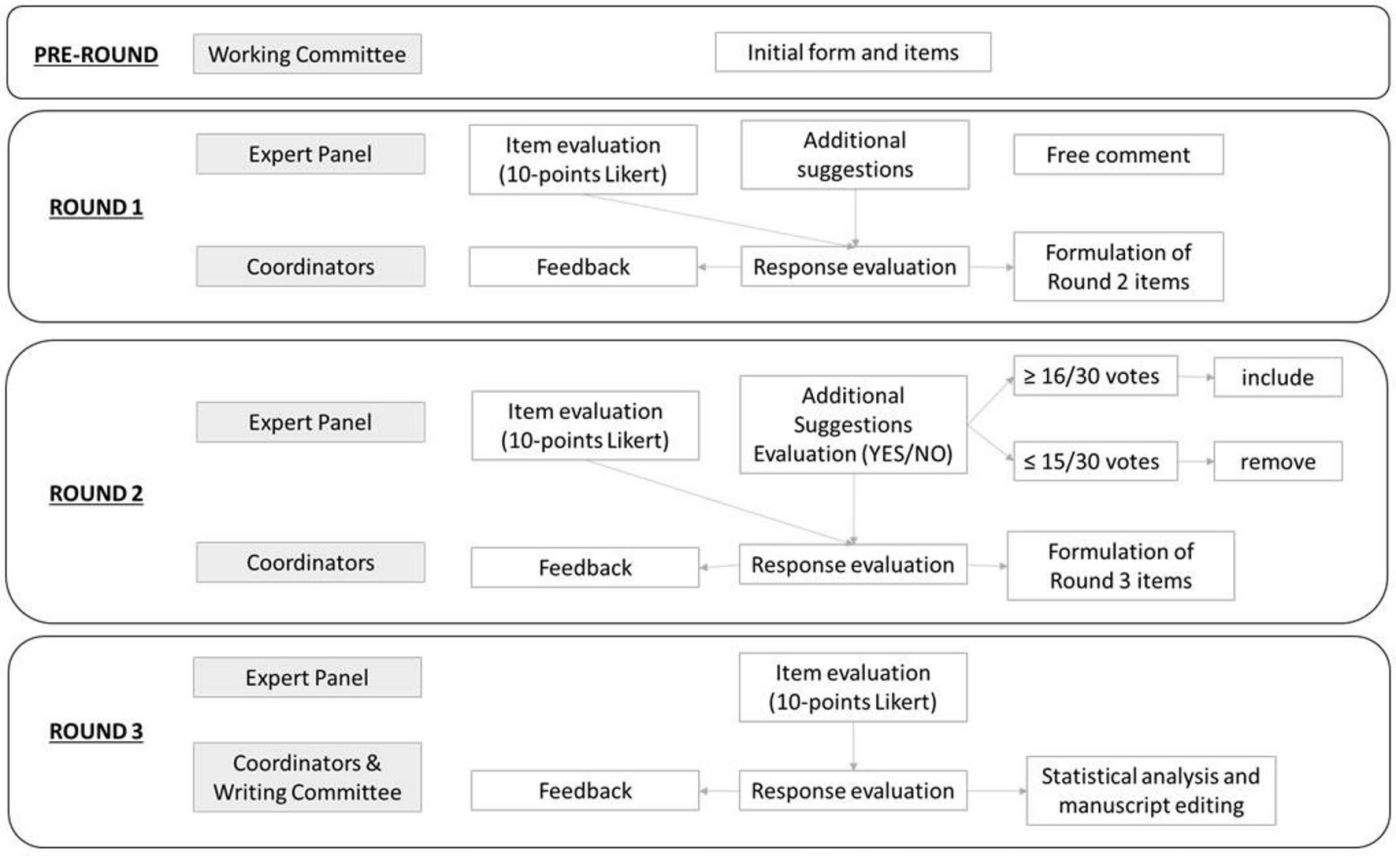
Number of items that reached a fixed threshold during round iteration

**Fig. 3:**
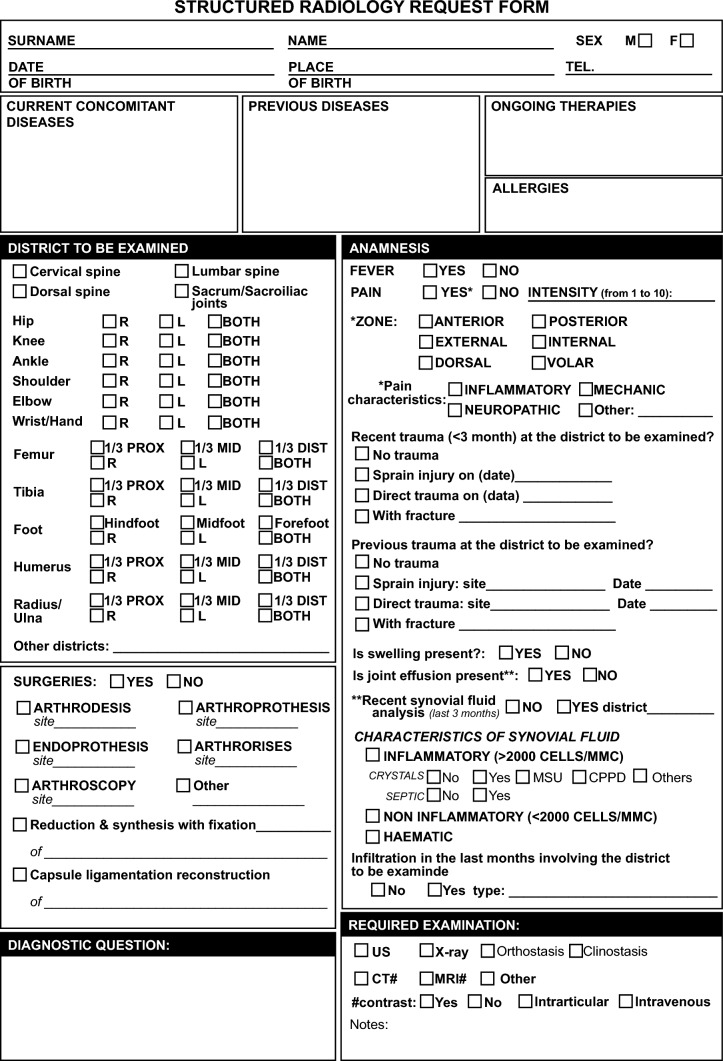
English version of the structured radiology request form

Round 2 consisted of 64 additional items to be voted on. Of these items, only six reached the threshold to be integrated into the form (≥ 16/30 votes) (Fig. 2). Round 3 consisted of 26 items to be voted on, and all items met the fixed validation thresholds with a high agreement (item medians range 9–10) and a sufficient consensus (IQR range 1–2). At the end of round 3, *p* was evaluated, which met our criteria for sufficiency response stability (> 0.05) among the expert panel for all items. Table 2 shows the final items and the result of Delphi iterations in terms of agreement, consensus, and stability obtained in round 3 in detail for each item. The final template in the English version is illustrated in Fig. 3. (The Italian version of the structured radiology request form is reported in Supplementary Material.)

**Table 2:** Final version of the items and results

Section	Item	Agreement (median)	Consensus (IQR)	Stability (*p*)
Patient personal information	Name Sex Place of birth Date of birth Phone number	10	1.5	0.572
Pathologic and pharmacologic history	Current diseases Prior diseases Drug therapies in place Allergies (specially to contrast medium)	10	1	0.672
Anatomical region	Precise identification of the district to be explored (multiple answers)	9	2	0.954
Clinical features	Evidence of fever Evidence of pain (Yes/No) Intensity of pain (1–10) Site of pain Pain characteristics (inflammatory, mechanical, neuropathic)	9	2	0.616
Trauma history	Recent traumatic injury (sprain, indirect, fracture) Prior traumatic injury (sprain, indirect, fracture)	9	2	0.360
Joint effusion information	Evidence of swelling Evidence of joint effusion Synovial effusion analysis Synovial effusion characteristic (inflammatory, non-inflammatory, hematic, presence of crystals)	9	2	0.986
Previous local treatments	Joint infiltration in the last month Previous surgery (yes/no) Type of previous surgery (multiple responses)	9	2	0.491
Clinical questions and type of radiological examinations	Clinical question Type of radiological examinations	10	1	0.227

*IQR* interquartile range

## Discussion

This paper presents the results of a Delphi consensus among interdisciplinary experts from SIRM, SIR and SIOT, focusing on the proper categorization of patients for radiological examinations of the musculoskeletal system, achieved through the development of a structured radiology request form.

Structured radiology request forms play a crucial role as a means of communication between referring physicians and radiologists. However, their significance often goes underestimated. Furthermore, there is a lack of standardization in these forms across different institutions, resulting in deficiencies in their completion [8]. Improperly filled or incomplete request forms are a global issue, leading to inappropriate imaging techniques and potential misinterpretation of results [16].

The request forms serve as both clinical and legal documents, completed by a referring clinician or their surrogate. They communicate the required procedure and its underlying reasons [6]. Guidelines from the Royal College of Radiologists stress the importance of completing these forms adequately and legibly to prevent misinterpretation. The radiologist holds ultimate responsibility for justifying the requested examination and assessing practical considerations related to patient radiation exposure, with the request form being the primary reference [7].

It is essential for physicians not to underestimate the importance of accurately and thoroughly completing request forms, as failure to do so can lead to medical errors or delays in essential treatment protocols.

The role of the clinical radiologist has evolved, shifting from a primary focus on imaging to a more patient-centered approach [8]. Radiologists now also play a crucial role in administering therapeutic interventions for various musculoskeletal disorders. To maximize efficiency, it is imperative that referring clinicians provide fully completed request forms.

These forms aim to present the clinical question that radiologists need to address. Some musculoskeletal conditions exhibit similar radiographic patterns, making comprehensive patient information crucial for accurate diagnosis. Missing information on a form can pose challenges for radiologists in narrowing down potential diagnoses associated with specific imaging patterns. This can lead to unnecessary inquiries, extended hospital stays, increased radiation exposure, and delayed patient management, raising costs for both patients and healthcare facilities. Insufficient or incomplete information on request forms significantly hampers a radiologist’s ability to assess the patient’s clinical condition efficiently. Multiple studies have shown a global deficiency in the completion of radiology request forms [6, 8, 11]. In this context, Jumah et al. conducted a study on widespread defects in request form submissions and proposed critical strategies to address these issues [17].

Compared to other proposed models, the form developed in this Delphi has the advantage of requiring minimal intervention with free-text input from the requester, as it is primarily based on checkbox completion. This aspect could promote accurate completion and interpretation of requests, as over 7% of requests made with freehand handwriting are not legible [18].

There are several limitations to mention. One limitation of this study is the inability to assess the potential benefits of implementing the intervention on a larger scale. Additionally, the study did not evaluate the appropriateness of the request form or the impact of interpretative comments on patient care. Subsequent investigations should focus on assessing the legibility and comprehensiveness of the structured radiology request form, alongside experiments to analyze its usability.

In conclusion, this paper presents the results through the development of a structured radiology request form deriving from a Delphi consensus among the members of the interdisciplinary expert panel regarding the proper categorization of patients for radiological examinations referred to the musculoskeletal system. The application of structured requesting on a large scale could be a method to promote better interaction between clinicians and radiologists, facilitate the diagnostic/differential pathway, and overall provide improvement in the care process.
